# A Review of SARS-CoV-2 Disease (COVID-19): Pandemic in Our Time

**DOI:** 10.3390/pathogens11030368

**Published:** 2022-03-17

**Authors:** Nasruddeen Al-Awwal, Ferris Dweik, Samira Mahdi, Majed El-Dweik, Stephen H. Anderson

**Affiliations:** 1 School of Natural Resources, University of Missouri, Columbia, MO 65211, USA; andersons@missouri.edu; 2 Department of Biomedical Engineering, University of Missouri, Columbia, MO 65211, USA; mmdmc5@mail.missouri.edu; 3 Cooperative Research, Lincoln University, Jefferson City, MO 65101, USA; samira.mahdi183@my.lincolnu.edu

**Keywords:** SARS-CoV-2 (2019-nCoV), COVID-19, MERS-CoV, outbreak, infection, symptoms, rapid detection

## Abstract

Development and deployment of biosensors for the rapid detection of the 2019 novel severe acute respiratory syndrome—coronavirus 2 (SARS-CoV-2) are of utmost importance and urgency during this recent outbreak of coronavirus pneumonia (COVID-19) caused by SARS-CoV-2 infection, which spread rapidly around the world. Cases now confirmed in February 2022 indicate that more than 170 countries worldwide are affected. Recent evidence indicates over 430 million confirmed cases with over 5.92 million deaths scattered across the globe, with the United States having more than 78 million confirmed cases and over 920,000 deaths. The US now has many more cases than in China where coronavirus cases were first reported in late December 2019. During the initial outbreak in China, many leaders did not anticipate it could reach the whole world, spreading to many countries and posing severe threats to global health. The objective of this review is to summarize the origin of COVID-19, its biological nature, comparison with other coronaviruses, symptoms, prevention, treatment, potential, available methods for SARS-CoV-2 detection, and post-COVID-19 symptoms.

## 1. Introduction

Coronavirus disease-19 (a.k.a. COVID-19) is a new disease caused by a coronavirus that is still under investigation concerning how it spreads. Coronavirus is spreadable. The current form of coronavirus disease, officially known as COVID-19, is the name of the disease caused by SARS-CoV-2 virus which was first reported in late December 2019 in Wuhan, China. Coronaviruses are a large family of viruses that are commonly found in infected people and many different animal species, including camels, cattle, cats, and bats. Coronaviruses belong to a group of enveloped viruses with a positive-sense, single-stranded RNA belonging to the β genus of Coronaviridae family and viral particles resembling a shape of a crown, hence the name corona. They belong to the order of Nidovirales and subfamily of Orthocoronavirinae, characterized by having an enveloped, non-segmented RNA. They possess a very large genome for RNA with viruses having the largest identified RNA genome up to 33.5 kilobases (kb) in size with a genome containing a 5′ cap structure along with a 3′ poly (A) tail, which allows for it to act as mRNA for translation of the replicase polyproteins. The gene encoding in the virus is the non-structural proteins (nsps) that occupy two-thirds of the genome, about 20 kb, as opposed to the structural and accessory proteins, which make up only about 10 kb of the viral genomes [1,2,3,4].

The SARS-CoV-2 virus belongs to betacoronaviruses category, such as MERS-CoV and SARS-CoV-1. All three of these viruses originated from bats [5,6]. The genetic sequences found in U.S. patients are similar to the ones that were obtained initially in China, suggesting a likely single, recent emergence of this virus from an animal reservoir [7]. Not often can animal coronaviruses infect people and then spread between people such as with Middle East respiratory syndrome (MERS), CoV, SARS-CoV, and now with this new virus (named SARS-CoV-2). It became evident that several domestic animals had previously tested positive for SARS-CoV-2 in some parts of the world including Hong Kong and Belgium, as well as in the New York City Zoo. However, there is not sufficient evidence that the COVID-19 can be transmitted to humans from these animals [8]. Recently, Pangolins (Manis sp) were reported to have been the prime suspects that could link host for SARS-CoV-2 even though the actual bridge host remains unknown [9].

## 2. Origin

The first human cases of COVID-19 were identified back in December 2019 in Wuhan City, China. At that time, the mode of transmission between humans was not clearly known. However, most infected people with SARS-CoV-2 reported exposure to a large seafood and wet animal market in Wuhan City, Hubei Province suggesting a potential zoonotic origin. Moreover, SARS-CoV, the virus which caused the SARS outbreak back in 2003, originated from animal reservoir (civet cats, a farmed wild animal) migrated to humans and then spread in the environment. In a similar way, it is thought that SARS-CoV-2 originated from animal species barrier and initially infected humans, but more likely through an intermediate host (that is, another animal species more likely to be handled by humans). This could be wild animal, or a domesticated wild animal and, as of now, has not been identified [10,11]. Older people and people of all ages with medical conditions or immune compromised, seem to be at higher risk of developing serious COVID-19 symptoms [8,9].

The advancement in technologies for the detection of SARS-CoV-2 variants played a critical role in obtaining reliable evidence about whether they are more transmittable, virulent, or more resistant to the available COVID-19 vaccines well before they spread throughout the globe. Genomic surveillance has increased the advantages in the field of next-generation sequencing. Therefore, researching and creating the availability of the whole genome data can aid in advancing the phylogenetic methods [12].

SARS-CoV-2 continuously undergoes mutation due to changes in the genetic code that usually occur during replication of its genome. These mutations have led to the formation of new variants that are genetically closely related with multiple variants being documented in the United States and globally during this pandemic. According to the CDC, there are about 12 lineage groups that inherited common ancestor. The common variants are the alpha (B.1.1.7, first isolated from United Kingdom and then Q lineages) with 50% increase transmission, may increase mortality; beta (B.1.351, first isolated in South Africa followed by descendent lineages, increased immune evasiveness, 50% increased transmission); delta (B.1.617.2) first isolated in India, the variant is almost twice as contagious as earlier variants and might cause more severe illness, may evade fully vaccinated people and increase rate of infection, possibly increases mortality; gamma (P.1, first isolated in Brazil/Japan, likely increased disease transmissibility and severity and then its descendent lineages); epsilon (B.1.427 and B.1.429, first isolated in California, 20% increased risk of transmissibility); eta (B.1.525); and iota (B.1.526, first isolated in New York, reported to likely increase rate of transmission). Other variants being monitored (VBM) are kappa (B.1.617.1), mu (B.1.621, B.1.621.1), and zeta (P.2) and the two most recent variants of concern (VOC) are the delta (B.1.617.2 and AY lineages) and omicron (B.1.1.529 and BA lineages, which are now present in more than 150 countries, it was first isolated in South Africa and presently, the most common strain in both UK and U.S. with over 50 mutations in spike protein). Currently, no SARS-CoV-2 variants were added to the list of variants of high concern (VOHC), as this would necessitate a notification to the World Health Organization (WHO) under the International Health Regulations [13,14,15,16,17].

## 3. Symptoms

COVID-19’s symptoms appear to be similar to those of Middle East respiratory syndrome (MERS), starting from 2–14 days of exposure to SARS-CoV-2, the symptoms include but are not limited to fever, tiredness, and dry cough. In some cases, patients may have aches and pains, nasal congestion, runny nose, sore throat or diarrhea, the new loss of taste or smell, repeated shaking with chills. These symptoms are usually mild and begin gradually. Some people (asymptomatic) become infected but do not develop any symptoms and don’t feel unwell [18,19,20,21]. More recently, a study of over 26,000 French adults was conducted wherein persistent physical symptoms were reported [22].

Some of the extreme COVID-19 symptoms were reported to involve tissue damaging by patients’ immune system and the study indicated an increase in the immune system molecule activities and Interleukin (IL-6) was reported to aid in cell regulations [23]. Long-term inflammation of lungs was reported to be one of the severe respiratory symptoms of COVID-19 which has been reported to be from the specific immune cells. A study conducted on 88 subjects with severe pneumonia caused by SARS-CoV-2 infection with most of the individuals having a high number of T-cells found in the lungs indicated that almost 70% of the alveolar macrophages which are typical immune cells situated in the lungs contained SARS-CoV-2 [24].

Almost all of the variants including delta and omicron cause similar COVID-19 symptoms that may stay over time. These symptoms include fatigue, shortness of breath or difficulty breathing, cough, joint pain, chest pain, memory concentration or sleep problems, dizziness, depression, or anxiety, fast or pounding heartbeat, worsened symptoms after physical or mental activities and this may cause muscle or body aches, pale, gray, or blue-colored skin, lips, or nail beds, depending on skin tone, inability to wake or stay awake, new loss of taste or smell, sore throat, congestion or runny nose, nausea or vomiting, and in some cases diarrhea and or gastrointestinal (GI) symptoms due to shedding of the virus. On the other hand, omicron is less likely to cause severe disease such as pneumonia that may require hospitalization but has a higher rate of spread with some evidence that fewer people lose their sense of taste and smell [25,26,27,28,29].

## 4. Biological Nature SARS-CoV-2 and Mechanism of Cell Entry

The SARS-CoV-2 membrane has the transmembrane (M) glycoprotein, the spike (S) glycoprotein, and the envelope (E) protein, and surrounds the flexible helical nucleocapsid. The viral membrane is unusually thick, probably since the carboxy-terminal region of the M protein forms an extra internal layer, as revealed by cryo-electron tomography [24]. Coronavirus enters in human cell through membrane ACE2 exopeptidase receptor, the mechanism of entry SARS-CoV-2 into the host cell is like the other coronaviruses such as SARS-CoV, Middle East respiratory syndrome coronavirus (MERS-CoV), and infectious bronchitis virus (IBV). SARS-CoV-2 becomes attached to the plasma membrane or use receptor-mediated endocytosis and fuses with endosomes based on the nature of the cells or tissues. The interaction between the virus-receptor and the cell allows for viral genetic material to be delivered to the host cell cytoplasm for replication, once delivered, the enzyme hemagglutinin-enterase (HE) dimer enhances spike protein (S)-assisted viral entry into the cell by binding to the host angiotensin converting enzyme 2 (ACE2) and the virus spreads throughout the mucosa. The envelope protein (E) facilitates the assembly and release of the virus. The membrane glycoprotein (M) provides structure to the virus and binds to the nucleocapsid protein (N), which binds to RNA helping to link the viral genome to the replicase-transcriptase complex, packaging the encapsulated genome into viral particles [30,31,32,33].

The close look at the isolate of a 2019-nCoV from a patient after performing genome sequencing of the 2019-nCoV in a study conducted by Lu et al and Hui et al in 2020 revealed that 2019-nCoV has 89.1% nucleotide similarity with CoVZC45 virus of bat origin and even exhibits 100% amino acid similarity in the nsp7 and E proteins. In another study involving nine patients, the data from the genome sequencing of the 2019-nCoV that were analyzed exhibited about 100% similarity in their identities [34,35].

SARS-CoV-2 continuously undergoes mutation due to changes in the genetic code that usually occur during replication of its genome. These mutations have led to the formation of new variants that are genetically closely related with multiple variants being documented in the United States and globally during this pandemic. According to CDC, there are about 12 lineage groups that inherited common ancestor. The common variants are the alpha (B.1.1.7 and Q lineages), beta (B.1.351 and descendent lineages), gamma (P.1 and descendent lineages), epsilon (B.1.427 and B.1.429), eta (B.1.525), iota (B.1.526), kappa (B.1.617.1), 1.617.3, mu (B.1.621, B.1.621.1), zeta (P.2) and the two most recent variants of concern delta (B.1.617.2 and AY lineages) and omicron (B.1.1.529 and BA lineages) [36].

## 5. Mode of Spread

The respiratory tract might not be the only route for the SARS-CoV-2 into the human body. SARS-CoV-1 is predominantly transmitted through direct or indirect contact with mucous membranes in the eyes, mouth, or nose [37], and this could be the case too with SARS-CoV-2 in contact with unprotected eyes. This can cause acute respiratory issues [38].

According to the CDC, SARS-CoV-2 (COVID-19) frequently spread from person-to-person who come in close contact (within about 2 m). This type of transmission occurs via respiratory droplets as the infected person coughs or sneezes. However, the virus has also been found in blood and stool, which raises questions about other possible routes of transmission [39,40]. On the other hand, the transmission of novel coronavirus to persons from surfaces contaminated with the virus has not been documented but recent studies indicate that asymptomatic people are likely to play a role in the spread of COVID-19. Recent evidence suggests that SARS-CoV-2 may remain viable from a few hours up to some days on surfaces based on the nature of the surface [41].

The new variant B.1.1.7 was first isolated in the United Kingdom and later was found circulating in Israel. A study conducted by Kustin et al., 2021 in evaluating the vaccinated and unvaccinated to compare the breakthrough infection. The comparison revealed that infections in partially immunized people were slightly more likely to be caused by B.1.1.7 as were those in unvaccinated people [42]. A Costa Rican PANGOLIN lineage B.1.1.389 with a spike (S: T11171) was reported to be the cause mutation T11171 in SARS-CoV-2 genomic sequence which was believed to be responsible for almost 30% of the cases that were reported in Costa Rica back in December of 2020 [43]. In another instance with the Costa Rican lineage, the B.1.1389 lineage happened to be transient as it began to disappear and gave room to more advantageous variants such as alpha and gamma. The mutation in S:T11171 that was found in Costa Rican lineage B.1.1.389 was believed to be a product of natural selection with some effects on the activity of the function and interaction of the spike protein [44].

The concept of co-infection (simultaneous infection of a host by multipleSARS-CoV-2 variants) cannot be overemphasized as there are recent studies that demonstrated genomic evidence of the existence of co-infection or within-host variation [45]. According to a study conducted by Chekuri et al. (2021). Patients with SARS-CoV-2 along with a non-influenza respiratory virus happen to have less severe disease and were more likely to be admitted but did not experience more severe effects than those infected with SARS-CoV-2 alone which could be due to the viral interference. Their study involved 306 SARS-CoV-2-positive patients with respiratory pathogen panel (RPP) and they observed severe COVID-19 effect in 111 (36.3%) patients in the SARS-CoV-2-only group and 3 (21.4%) patients in the co-infected group, even though the result was not significantly different [46]. Molina-Mora et al. (2022) have utilized two different approaches one being the metagenomic pipeline which is based on the inference of multiple fragments such as amplicon sequence variant (ASV-like) from sequencing data and a custom SARS-CoV-2 database to identify the concomitant presence of divergent SARS-CoV-2 genomes which were used to analyze 1021 cases of COVID-19 from Costa Rica to investigate the possible occurrence of co-infections and compared this with another strategy which was based on the whole-genome (metagenome) assembly; the results indicated an accuracy of about 96.2% with for ASV-like and 46.2% for the whole—genome assembly strategy [47].

The emergence of the new variants has led to more research in order to correctly characterize the nature of the mutant virus in terms of changes in transmission, severity of the disease, symptoms, mortality rate, and effectiveness of the available vaccines [48].

## 6. Prevention

Cleaning of either visibly dirty or non-dirty surfaces followed by disinfection is the best practice to minimize the spread of COVID-19 and other viral respiratory illnesses in households and community settings. It is not certain how long the air inside a room occupied by someone with confirmed COVID-19 remains potentially infectious. Facilities have considered factors such as the size of the room and the ventilation system design (including flowrate [air changes per hour] and location of supply and exhaust vents) in order to minimize contacts. Taking measures to improve ventilation in an area or rooms where people with illness or suspected to be exposed with COVID-19 will help shorten the time it takes respiratory droplets to be removed from the air and social distancing too, has been reported to be effective in curtailing the spread of SARS-CoV-2. The use of personal protective equipment (PPE) including masking and general body hygiene in both health care settings and homes is of uttermost importance in reducing the viral spread and quarantining individuals that were exposed as a way to break the chain of the spread [49].

Several SARS-CoV-2 vaccines are now in circulation and there are more additional candidates in the pipeline. All of this is dependent upon the technological platforms being used in the vaccine production with each platform having its own advantage. Inactivated platform is the well-established manufacturing process used to produce purified whole SARS-CoV-2 components, this is the type of technology used by the Chinese scientists to produce CoronaVac (PicoVacc) and BBIBP-CorV. The other two newest platforms are the Nucleic acid and the viral vector-based vaccines. The nucleic acid platforms are usually rapid and having low cost of manufacturing and they can either be lipid-encapsulated mRNA such as in the mRNA1273 vaccine produced by the United States based company, Moderna and BNT162b1 from BioNTech/Fosun Pharma/Pfizer or self-amplifying mRNA type that was used to produce LNP-nCoVsaRNA vaccine by Imperial College London. The viral vector on the other hand, it involves robust cellular and humoral vaccine immunity, and this was the technology being used in the production of the Human Adenovirus type 5 and 26 (Ad5-nCoV and Ad26.COV2-S) from the Japanese Pharmaceutical companies and it was the same technology used in the production of chimpanzee adenovirus (ChAdOx1 nCoV19 by AstraZeneca, University of Oxford [50,51,52,53,54,55,56,57,58,59,60,61,62,63,64,65,66]. Some other scientists are working on vaccine candidate designs, Samad et al. (2022) worked on determining the immunodominant T- and B-cell epitopes using the SARS-CoV-2 spike glycoprotein and they were able to design a vaccine construct using four potential epitopes from three epitopes namely the cytotoxic T-lymphocytes, linear B-lymphocyte and helper T-lymphocyte epitopes thereby designing a vaccine that was immunogenic, antigenic and non-allergenic with suitable physicochemical properties and higher solubility [67].

A wide range of trials for COVID-19 vaccines were put in place to assess the long-term ability of the vaccines in preventing the spread of the virus among individuals even if they show no symptoms. Hall et al. 2021 claimed that Pfizer and BioNTech vaccines were 70% effective at preventing infections in both symptomatic and asymptomatic individuals after three weeks of getting the first dose in the UK and the effectiveness grew up to 85% after the second dose. The study comprised of 23,000 health-care workers but this research has not been peer reviewed yet [68].

A clinical trial that was conducted by Thompson et al., 2021 in 8 U.S. locations and has indicated that the Moderna and Pfizer–BioNTech mRNA-based vaccines are highly effective at protecting people from illness caused by SARS-CoV-2 by 80 to 90%. A study conducted on nearly 4000 people whose work puts them at high risk of infection, the individuals were vaccinated in mid-December 2020 and mid-March 2021 (participants were considered fully immunized) and they were monitored by swabbing their noses for the viral sample collection once a week for 13 weeks. The result indicated that there was a 90% effectiveness at protecting people against the infection and for the single dose, it was about 80% effectiveness but with all of these, it has been cautioned as it is hard to evaluate the effectiveness of the vaccine against the infection with high precision [69].

Another clinical trial that was carried out on 22,000 people in Russia using a modified cold virus showed 91% effectiveness against symptomatic COVID-19. The Sputnik V vaccine (Gam-COVID-Vac) developed at the Gamaleya National Center of Epidemiology and Microbiology in Moscow contains two kinds of adenovirus (typically cause mild illnesses such as common cold), each carrying genetic instructions for the spike protein that SARS-CoV-2 uses to latch onto host cells [70].

The efficacy of the vaccines against emerging SARS-CoV-2 variants of concern, including the B.1.351 (501Y.V2) variant first identified in South Africa was investigated by Madhi et al. (2021), Of the 25 participants who were tested by pseudovirus and live-virus neutralization assays against the original D614G virus and the B.1.351 variant by means of collecting the participants serum after the second dose, a two-dose regimen of the ChAdOx1 nCoV-19 vaccine did not show protection against mild-to-moderate COVID-19 symptoms due to the B.1.351 variant, suggesting that the vaccine is less effective against the variant B.1.351 than against other variants [71]. From 14 December 2020, through 22 February 2022, there were more than 550 million doses of COVID-19 vaccines that were dispensed in the United States, the vaccine adverse event reporting system (VAERS) has officially received 12,612 preliminary reports of death (0.0023%) among people who received a COVID-19 vaccine. Other side effects such as cerebral venous sinus thrombosis with thrombocytopenia, a serious condition involving blood clotting, were reported in some parts of Europe following receipt of the ChAdOx1 nCoV-19 vaccine (Oxford/AstraZeneca). In the US, the Ad26.COV2. S COVID-19 vaccine (Janssen/Johnson & Johnson), which uses a human adenoviral vector, received Emergency Use Authorization (EUA) on 27 February 2021, and there were six cases of cerebral venous sinus thrombosis reported by April 2021 [72,73].

## 7. Methods of Detection

Early detection of COVID-19 is very critical for the disease control. Detection of COVID-19 is carried out through biomarkers by targeting biological markers without affecting the body. Structural proteins including spike (S) and nucleocapsid (N) are used as biomarkers in detection [74].

Several detection methodologies are available ranging from serological methods which include enzymes based on their binding affinity against SARS-CoV-2 and coated antigen plates containing both primary and secondary antibodies leading to the production of fluorescence that can be analyzed, the method known as enzyme link immunosorbent assay (ELISA) which is rapid and less expensive or chemiluminescence immunoassay (CLIA) which utilizes chemical probes that can produce light emission through chemical reaction with the labeled antibody against SARS-CoV-2, this is sensitive and rapid compared to ELISA but costlier. Another way to detect the SAR-CoV-2 is via molecular methods such as real-time reverse transcript polymerase chain reaction (RT-PCR) technique which involves conversion of RNA of COVID-19 to cDNA through transcriptase enzyme followed by real-time PCR for the DNA amplification. This is a gold standard method. It is sensitive and expensive with detection limit of about 250 genomic copies/mL. Real-time loop-mediated isothermal amplification (RT-LAMP) is another type of molecular method which is similar to RT-PCR in terms of RNA conversion but does not require thermocycler, making it time efficient and capable of detecting as low as 1.5 genomic copies/µL. Then, there is amplicon-based metagenomic sequencing which is used to amplify certain regions in gene by PCR, subsequently evaluated using next-generation sequencing (NGS). The results are compared against the database, and this method can diagnose different coronavirus strains. Nucleic acid hybridization using microarray is another molecular method being used to detect SARS-CoV-2. It is a similar technique to RT-LAMP but after converting the RNA to cDNA, the cDNA is added to a well containing oligonucleotides that is specific to the virus followed by purification of the complex via washing the hybridized virus and measuring its signal. Both the amplicon-based metagenomic sequencing and the nucleic acid hybridization techniques are sensitive and expensive. Other methods for detecting SARS-CoV-2 include the point-of-care detection of COVID-19 which is based on either lateral flow or biosensing which mostly are based on nanotechnology coupled with antibodies (IgM and IgG) with some lines as gold nanoparticles and others as clustered gold on account of plasmon band coupling. The latest technique for the detection SARS-CoV-2 is the clustered regularly interspersed short palindromic repeats (CRISPR) technology via coupling with fluorescence, the detection time is within one hour using the SHERLOCK (specific high sensitivity enzyme reporter unlocking) technique and can have a lower cost of analysis on an industrial scale when compared to qRT-PCR which is also prone to false negative results. However, we must bear in mind that the CRISPR technology has its own limitations too since it is pretty much new technology that is still being worked on to understand more of it. The advancement in the technological world has made almost all of these methods achievable within 24 h or even less [75,76,77,78,79,80,81].

Many studies were conducted to monitor and analyze the progress of pneumonia in certain organs; lungs, liver and heart using X-ray with an artificial intelligence model with deep learning system that could differentiate between viral pneumonia and other types of pneumonia and absence of pneumonia based on computer X-ray images. Tao et al. used chest computed tomography (CT) imaging to diagnose COVID-19 and indicated its high reliability with a very sensitive result of 98% [82,83].

Diagnosis time is a critical element in the process towards treatment, and the need for faster, high accuracy detection still exists. The field of biosensing offers new modes of detection as researchers explore the use of nanomaterials to enhance the limit of detection (LOD) which can speed up the diagnosis time. One example of these new lines of COVID-19 biosensors is a graphene-based nanosensor currently being developed by the Zhang lab [84]. 

## 8. Treatment

At the beginning of the pandemic, there was no any specific antiviral drug that had been proven to be effective against SARS-CoV-2 which was available in the market. However, a study conducted by Cao et al., demonstrated that remdesivir (GS-5734), a broad-spectrum nucleoside analogue antiviral prodrug was found to have some effects on pathogenic animal and human coronaviruses including SARS-CoV-2, with in-vitro studies showing it can inhibit SARS-CoV-2, SARS-CoV and MERS-CoV replications. However, the medication was an experimental drug and when in combination of baricitinib, it shaved off one day of the recovery of the COVID-19 patients [85,86,87,88].

There were a lot of controversies among scientist on the efficacy of the hydrochloroquine (HCQ). This was approved in some countries such as France, China, and Korea with recommendations for it to be combined with other drugs such as lopinavir, ritonavir, α-interferon, and umifenovir (and in many cases with azithromycin (AZ)). Hydroxychloroquine (HCQ) was found to be efficient in clearing viral nasopharyngeal carriage of SARS-CoV-2 in COVID-19 patients in only three to six days (in most patients). A significant difference was observed between hydroxychloroquine-treated patients and controls at earliest on a day three post-inclusion. These results demonstrate great importance due to the study which has shown that the mean duration of viral shedding in patients suffering from COVID-19 in China was 20 days (even 37 days for the longest duration) and retinopathy is one of the major adverse reactions of long-term therapy with HCQ which has shown about 90% efficacy [89,90].

There are other anti-inflammatory drugs that have been reported to have lowered the need for an invasive ventilation. A clinical trial with 21,550 patients out of which 4116 were enrolled in a RECOVERY trial program in which they were receiving oxygen and had evidence of systemic inflammation, 2022 patients were treated with tocilizumab, a drug designed to help with the immune response, and 729 (35%) of the 2094 patients were kept under usual care were dead within 28 days. The trial proved administering the tocilizumab was associated with a greater probability of discharge from hospital within 28 days (57% vs. 50%), and hence the drug has cut the need for invasive ventilation [91]. In another study conducted by Gordon and colleagues, the same drug has reduced the death rate from nearly 36% in the control group to about 28% [92].

Another oral drug EIDD-2801(an oral broad-spectrum antiviral agent) was used based on the animal model has demonstrated significant reduction of SARS-CoV-2 in the lungs. It is currently in the late clinical trial stage and has been reported to inhibit SARS-CoV-2 replication in vivo, therefore being considered as having potential for the prevention and treating COVID-19 [93].

The recent technological advancement has yielded computational drug repurposing campaign among researchers in order to identify potential inhibitors of the main protease of the SARS-CoV-2, the spike angiotensin-converting enzyme 2 (ACE-2). Some of the compounds already in the clinical trial stage are being assay for their antiviral activity. The computational workflow involves analyzing set of inhibitors from the DrugBank database to select the required ligands, followed by molecular docking calculation in the active sites of the ligands to validate the docking protocol, then optimizing the protein-ligand interaction and looking at the geometries and binding free energy estimations then evaluating the protein-ligand complementarily through visual inspection, then analysis of the activity annotations and therapeutic indications of the potential candidate compounds and finally the candidate selection [94].

There are other drugs that are worth exploring for the potential COVID-19 treatment as preventing ACE-2 which aid SARS-CoV-2 to penetrate the cells is of paramount importance in treating COVID-19. Lazniewski et al. (2021) worked on drug repurposing involving zafirlukast which is used for treating asthma by blocking a leukotriene receptor and simeprevir which is a protease inhibitor used for hepatitis C treatment. Both drugs have average binding affinity of −22 kcal/mol for spike proteins originating from various lineages, hence potential in exploring in vitro and in vivo against SARS-CoV-2 [95].

Pokhrel et al. (2021) utilized computer aided drug design (CADD) to produce four potentially natural ACE2 inhibitors for the treatment of COVID-19. They were able to identify four natural compounds Amb17613565, Amb6600091, Amb3940754, and Amb21855906 that can potentially inhibit the activity of ACE2 and result in blocking the entry of SARS-CoV-2 into the human host cell. All of the four candidates have no likeness to synthetic drugs, and they all have high water solubility, and all have high gastrointestinal absorption regarding their pharmacokinetics except Amb6600091 which has low absorption [96].

As of 2022, three therapeutic antibodies have been issued through the Emergency Use Authorization (EUA) by the United States Food and Drug Administration (U.S. FDA). One is a cocktail pair with bamlanivimab along with etesevimab with data that suggest it may lead to a decline in virus levels in patients. The second uses a cocktail pair casirivimab and imdevimab. The last is a single antibody from sotrovimab with its clinical trial showing an 85% reduction in hospitalization or death. Currently, only emergency use authorization was approved by the US FDA for Paxlovid (nirmatrlvir tablets and ritonavir tablets) loaded to stop the virus from replicating [97].

## 9. Post COVID-19 Conditions

Most people who been exposed to SARS-CoV-2 usually recover completely within a few weeks. But while some people (even those who had mild versions of COVID-19) tend to continue to experience symptoms after their initial recovery, this is usually referred to as post-COVID-19 syndrome or post-COVID-19 conditions. The conditions usually persist for more than four weeks with older people and people who are immunocompromised are the most prone to experience these conditions.

The recent reinfection that has been occurring at irregular intervals has been reported to be associated with the rapid decline in antibody levels, which raises serious concern that the immunity to SARS-CoV-2 could decrease within weeks of recovery from the infection with immune system’s memory lingering for about six to eight months in most people. There should large amount of data in order to make conclusions about protective immunity based on quantifying SARS-CoV-2 circulating antibodies, even though immune memory is the basis for understanding the long-term protective immunity [98].

A study conducted by Carfi et al. (2020) reported a post-acute symptom that were established in an outpatient department in Italy. The patients were monitored after the appearance of the first symptoms and out of 143 patients, about 88% got a persistence symptom. Montenegro and his colleagues studied 579 patients using standardized questionnaire in three different primary health care centers in Spain and collected information on symptoms and found that the most frequent persistent symptoms were fatigue (44.6%), smell impairment (27.7%) and dyspnea (24.09%) and that the prevalence was higher in women than men with 15.63% and 13.06%, respectively. The neurological disorders were reported to have been a part of post-COVID-19 condition, with some symptoms present after three months from the onset of acute COVID-19 disease. These were reported to have included cognitive dysfunction involving memory concentration, attention disorder, and in some cases sleep deprivation, which appeared to be the key features of post-COVID-19 syndrome. Normally, sleep deprivation and depression are the most common symptoms over time [99,100,101,102].

The respiratory system is believed to be one of the most involved in COVID-19 exposure among other systems and helps in diagnosing the extended post COVID-19 symptoms such as cough and shortness of breath. Complications such as lung functioning abnormalities, cough, pneumonia, pulmonary embolism, and other related conditions were reported to have been monitored with equipment such as over time pulse oximetry, chest X-ray, high-resolution computed tomography of the chest, and pulmonary angiogram over time [103,104,105]. A lot has not been known about how COVID-19 is affecting people over time since research is still ongoing. It is recommended that doctors should closely monitor people who have had COVID-19 to see how their organs are functioning after recovery.

## 10. Conclusions

SARS-CoV-2 is transmitted primarily through the respiratory tract. Additionally, close contact (person-to-person) and contact with contaminated surfaces are potential sources for contracting COVID-19. Current available diagnostics being used were developed by retrofitting existing methods to the current SARS-CoV-2 virus. Each of them has their own trade-offs in terms of specificity as well as sensitivity. However, one significant problem with these methods is that the detection results are received after the person has been infected for many days. This means the individual could have spread the virus during that time while the symptoms become progressively more severe. Research and development of a diagnostic which could detect the presence of SARS-CoV-2 earlier on would be greatly beneficial. Another missing area of diagnostics is on the environmental side. If environmental detection of this virus could occur, it could aid in prevention of infection significantly and reduce the number of patient tests needed.
